# Aphid secondary symbionts do not affect prey attractiveness to two species of predatory lady beetles

**DOI:** 10.1371/journal.pone.0184150

**Published:** 2017-09-07

**Authors:** Jennifer L. Kovacs, Candice Wolf, Dené Voisin, Seth Wolf

**Affiliations:** 1 Biology Department, Spelman College, Atlanta, Georgia, United States of America; 2 Larner College of Medicine, University of Vermont, Burlington, Vermont, United States of America; 3 Neuroscience Institute, Georgia State University, Atlanta, Georgia, United States of America; Universidade Federal de Vicosa, BRAZIL

## Abstract

Heritable symbionts have been found to mediate interactions between host species and their natural enemies in a variety of organisms. Aphids, their facultative symbionts, and their potential fitness effects have been particularly well-studied. For example, the aphid facultative symbiont *Regiella* can protect its host from infection from a fungal pathogen, and aphids with *Hamiltonella* are less likely to be parasitized by parasitic wasps. Recent work has also found there to be negative fitness effects for the larvae of two species of aphidophagous lady beetles that consumed aphids with facultative symbionts. In both species, larvae that consumed aphids with secondary symbionts were significantly less likely to survive to adulthood. In this study we tested whether adult *Harmonia axyridis* and *Hippodamia convergens* lady beetles avoided aphids with symbionts in a series of choice experiments. Adults of both lady beetle species were as likely to choose aphids with symbionts as those without, despite the potential negative fitness effects associated with consuming aphids with facultative symbionts. This may suggest that under natural conditions aphid secondary symbionts are not a significant source of selection for predatory lady beetles.

## Introduction

Close associations between eukaryotic organisms and prokaryotic symbionts are ubiquitous across the tree of life. These relationships can be detrimental to the host, as in parasitism, neutral for both parties, as in commensalism, or beneficial to the host and the symbiont, as in mutualism [1]. Host-symbiont mutualisms have been particularly well-studied in insects, and one of the most thoroughly studied has been that of the pea aphid *Acyrthosiphon pisum* and its single obligate and multiple facultative bacterial symbionts. All *A*. *pisum* individuals harbor the obligate nutritional symbiont *Buchnera aphidicola* in specialized cells known as bacteriocytes. Additionally, approximately 80% of *A*. *pisum* individuals carry one or more facultative symbionts [2]. Currently seven facultative symbionts have been identified in different populations of *A*. *pisum* [3].

Three of these facultative symbionts, *Regiella insecticola*, *Hamiltonella defensa*, and *Serratia symbiotica*, have been the focus of multiple studies seeking to identify potential benefits of infection to the aphid host as well as understand the ecological consequences of protective symbioses [4–7]. For example, *Regiella* can allow for its pea aphid host to utilize white clover as its host plant [8] and increase its host’s resistance to a fungal pathogen [9]. *Serratia* and *Hamiltonella* can both help their hosts survive bouts of high temperature heat shock [10,11]. Additionally, both *Hamiltonella* and *Serratia*, as well as at least one strain of *Regiella*, have been found to protect their hosts against parasitism by parasitoid wasps [12].

In addition to these fitness effects, aphid facultative symbionts can also affect the survival of aphid predators. Recent work by our group found that the consumption of aphids harboring facultative symbionts by the larvae of two lady beetle species can affect larval survival, pupal survival, development time, and adult weight in females. Specifically, in the convergent lady beetle *Hippodamia convergens*, larvae that consume pea aphids with either the *Hamiltonella* or *Serratia* symbionts were significantly less likely to survive to pupation [13]. Adult females fed aphids with the *Hamiltonella* and *Serratia* symbionts as larvae also weighed significantly more than those that had been fed symbiont free aphids [13]. While in the Asian multi-colored lady beetle *Harmonia axyridis*, we found that larvae that consumed aphids with either the *Serratia* or *Regiella* symbiont were significantly less likely to survive their larval or pupal stages [14]. *Harmonia axyridis* larvae fed aphids with the *Regiella* symbiont had longer larval and pupal development times, and females that did survive to adulthood, weighed significantly less than those fed symbiont free aphids. Overall, these findings suggest that aphid symbionts can have a significant impact on the survival and fitness of multiple aphid predators. [13]

In our previous work, we found no significant difference in feeding rates of adult *Hi*. *convergens* beetles fed aphids with or without symbionts [13]. However, the findings of fitness effects in both predatory lady beetle species *Hi*. *convergens* and *Ha*. *axyridis* led us to ask whether lady beetles avoid consuming aphids with secondary symbionts if given the option. We predicted that lady beetles when given a choice would avoid eating aphids with symbionts due to potential effects on survival. We also predicted that female *Hi*. *convergens* lady beetles may actually prefer aphids harboring *Regiella* or *Hamiltonella* symbionts due to an increase in adult weight in this species, which could possibly increase female fecundity. In this study, we designed a series of choice experiments to see if *Ha*. *axyridis* and/or *Hi*. *convergens* demonstrated a preference or avoidance for aphids harboring the *Serratia*, *Regiella*, or *Hamiltonella* over aphids without secondary symbionts.

## Materials and methods

We performed several sets of choice experiments to determine whether adult lady beetles exhibited a preference for symbiont free aphids over those harboring three different facultative symbionts (*Serratia symbiotica*, *Regiella insecticola*, or *Hamiltonella defensa)*. Adult male and female *Ha*. *axyridis* and *Hi*. *convergens* were used in these experiments.

### Acyrthosiphon pisum

Pea aphids (*Acrythosiphon pisum*) from genetically identical asexual aphid lineages harboring either no facultative symbionts (aphid line 5AO), the facultative symbiont *Serratia symbiotica* (5AR), *Hamiltonella defensa* (5AT), or *Regiella insecticola* (5AU) were used in all choice experiments. All four aphid symbiont lineages (5AO, 5AR, 5AU, and 5AT) were established from the same naturally uninfected 5A clone (collected in Madison, WI, USA, June 1999). Facultative symbionts were introduced to the 5A clone through microinjection of body fluids containing symbionts (5AR & 5AU, [12], 5AT, [15]). Prior to starting the experiment, lines were screened for the respective facultative symbionts using qPCR ([16], unpublished data). In addition, *H*. *defensa* is associated with a phage, APSE, that is known to affect the *Hamiltonella*-conferred phenotype. We used PCR to confirm the presence of APSE in our Hamiltonella-infected line ([16], unpublished data). Aphids were reared on fava seedlings (*Vicia faba* L.) at 20°C with a light regime of 18:6 Light:Dark prior to being used in the choice experiments. Though some mutations between the sub-clonal lines may be present [17,18], by using sub-clonal aphid lineages we were able to control for aphid genotype differences that may affect beetle feeding behavior. Therefore any differences observed in aphid preference or avoidance would be due solely to the absence or presence of the aphid symbiont.

### Choice chambers

To test for aphid preference, each lady beetle was placed in the middle of a straight, rectangular, polystyrene structure that was 8 cm long X 1.5 cm tall X 1.5 cm wide. The entire choice chamber was enclosed to prevent the ladybeetle from escaping or flying away. On each end of the chamber a small cage in which aphids were placed was attached. The aphid cages were polypropylene tubes that were 1 cm high with a 0.5 cm diameter. From each aphid cage a 0.5 cm wide x 0.6 cm high rectangle was removed from the part of the tube facing the lady beetle. This rectangle was covered with a single layer of grade #40 cheesecloth (9.5 x 8 threads per cm). This mesh allowed the beetle to see the aphids as well as come into contact with the aphid, but did not allow them to grab, bite, or eat the aphid. A preliminary series of 12 trials were run in which one chamber contained 5 symbiont free aphids and the other was empty. In two of the trials the adult *Ha*. *axyridis* did not make a choice. In eight trials the lady beetle chose the cage with aphids, and in two trials, it chose the empty cage (*χ*^2^ = 3.85, *p* = 0.05). Based on these preliminary results, we determined that the lady beetles could locate aphids in our choice chambers.

In all of our experimental trials one mesh cage contained symbiont-free aphids (5AO) and the other cage had aphids harboring one of three facultative symbionts; *Serratia* (5AR), *Regiella* (5AU), or *Hamiltonella* (5AT). The lady beetle then had the option to go towards either end of the chamber. If the lady beetle went in either direction and ended at the mesh cage and remained there for 45 seconds, the direction of the lady beetle was recorded. Trials were run for 15 minutes or until a choice was made. The type of aphid (symbiont harboring or symbiont free) was switched between the left and right side between trials. This was done to control for any potential directional preference or other unmeasured variables that may have affected lady beetle behavior. The symbiont free aphid was always placed in the same “symbiont-free aphid chamber” for every trial, though that chamber could be physically switched from one side of the chamber to the other by turning the chamber around between trials. Different chambers were built to test for each aphid symbiont type. This was done to control for any cross-contamination from volatile chemicals released by the aphids with symbionts that would allow for discrimination by the lady beetles between aphids with symbionts and those without.

### Harmonia axyridis

Adult lady beetles (*Ha*. *axyridis*) were collected at Spelman College in Atlanta, Georgia, USA. Adult lady beetles were not fed prior to the experiment. Lady beetles were kept in individual test tubes and maintained at 4°C with a light regime of 16:8 Light:Dark. Before each set of experiments was carried out the lady beetles were allowed to warm up for 2 hours at room temperature. Sexing of the lady beetles was performed using dimorphic features of the distal margin of the final abdominal sternite [19].

Ten adult *Ha*. *axyridis* beetles, five male and five female, were used in a total of 200 trials run over a ten day period. Beetles were used in two trials each day, and all trials with the same beetle were conducted at least three hours apart. One hundred and thirty-four of these trials resulted in a choice by the focal lady beetle, and these trials were the focus of our analyses. Seventy of these trials used one aphid per cage, and sixty-four trials used five aphids per cage. Fifty-two trials (29 with one aphid and 23 with five aphids) were run with *Serratia* infected aphid / symbiont free aphid choices. Fifty-three trials (26 with one aphid and 27 with five aphids) were run with *Regiella* infected aphid / symbiont free aphid choices. Twenty-nine total trials (15 with one aphid and 14 with five aphids) were run with *Hamiltonella* infected aphid / symbiont free aphid choices.

### Hippodamia convergens

Adult lady beetles (*Hi*. *convergens)* were obtained from Carolina Biological Supply. Upon receipt adults were not fed and were maintained as described for adult *Hi*. *convergens* (see above). Adults used in the choice experiments were sexed at the end of the experiments [19]. *Hippodamia convergens* choice experiments used the same experimental choice chamber setup as those described in the *Ha*. *axyridis* choice experiments. All *Hi*. *convergens* choice experiments used one aphid per mesh cage and were run for 15 minutes or until a choice was made. The side with the symbiont harboring aphid was physically switched after each round, and different choice chambers were used for each aphid symbiont type to avoid cross-contamination, as was done in the *Ha*. *axyridis* trials and is described above.

A total of 60 *Hi*. *convergens* choice trials in which an individual made a choice were completed. Twenty trials were recorded for each aphid-harboring symbiont, i.e. 20 *Serratia* infected aphid / symbiont free aphid choices, 20 *Regiella* infected aphid / symbiont free aphid choices, and 20 *Hamiltonella* infected aphid / symbiont free aphid choices. Thirty individuals were used in the choice experiments; 16 females and 14 males. On average each *Hi*. *convergens* adult was used in one trial for each symbiont type. No *Hi*. *convergens* beetles were used in more than one trial in a single day.

### Statistical analyses

In both species for each symbiont type, *χ*^2^ analyses were used to determine whether the number of trials in which the lady beetle chose the symbiont free aphid or the aphid harboring the focal symbiont was significantly different than the 50% that would be predicted by random chance alone. For each lady beetle species, the data was first pooled to analyze all symbiont-infected aphids together versus aphids lacking secondary symbionts. Data were then broken down further to investigate each symbiont type, and in the case of *Ha*. *axyridis* whether the number of aphids used in a trial impacted the outcome. We tested whether there was a preference for left or right using *χ*^2^. We also used *χ*^2^ analyses to determine whether males and females choose differently, and finally, for *Ha*. *axyridis* we were able to look at individual preferences for both aphids and direction using *χ*^2^.

Post-hoc power analyses were conducted using the software package, GPower2 [20]. For each species of lady beetle, the total number of trials, as well as the number of trials performed for each aphid symbiont type were used as the sample size for the statistical analyses. The recommended effect sizes for *χ*^2^ were as follows: small (effect size = 0.1), medium (effect size = 0.3), and large (effect size = 0.5; [21]). The alpha level used for the analysis was *p* < 0.05, and a power of 0.80 or greater was considered adequate to detect a significant effect at each effect size level. Our post-hoc power analyses revealed that a sample size of 785 trials would be necessary to detect small effects of symbiont, 87 trials to detect medium effects, and 31 trials to detect large effects. Therefore, our *Ha*. *axyridis* trials for all symbionts combined had adequate statistical power to detect effects at the medium effect level, the *Serratia* and *Regiella* trials had more than adequate statistical power to detect large effects (power = 0.96). Our sample size for the *Hamiltonella* trials had a power of 0.77, just under the 0.80 cutoff, for detecting large effects. For *Hi*. *convergens* when all trials were combined to compare symbiont free to all symbionts, there was more than adequate statistical power to detect large effects (power = 0.97), but only had a power of 0.64 for detecting medium effect sizes. The sample size of 20 trials for each symbiont type for *Hi*. *convergens* had a power of 0.61 for large effects, and therefore non-significant results for these trials may be the result of a Type II error. [21]

## Results

**Fig 1:**
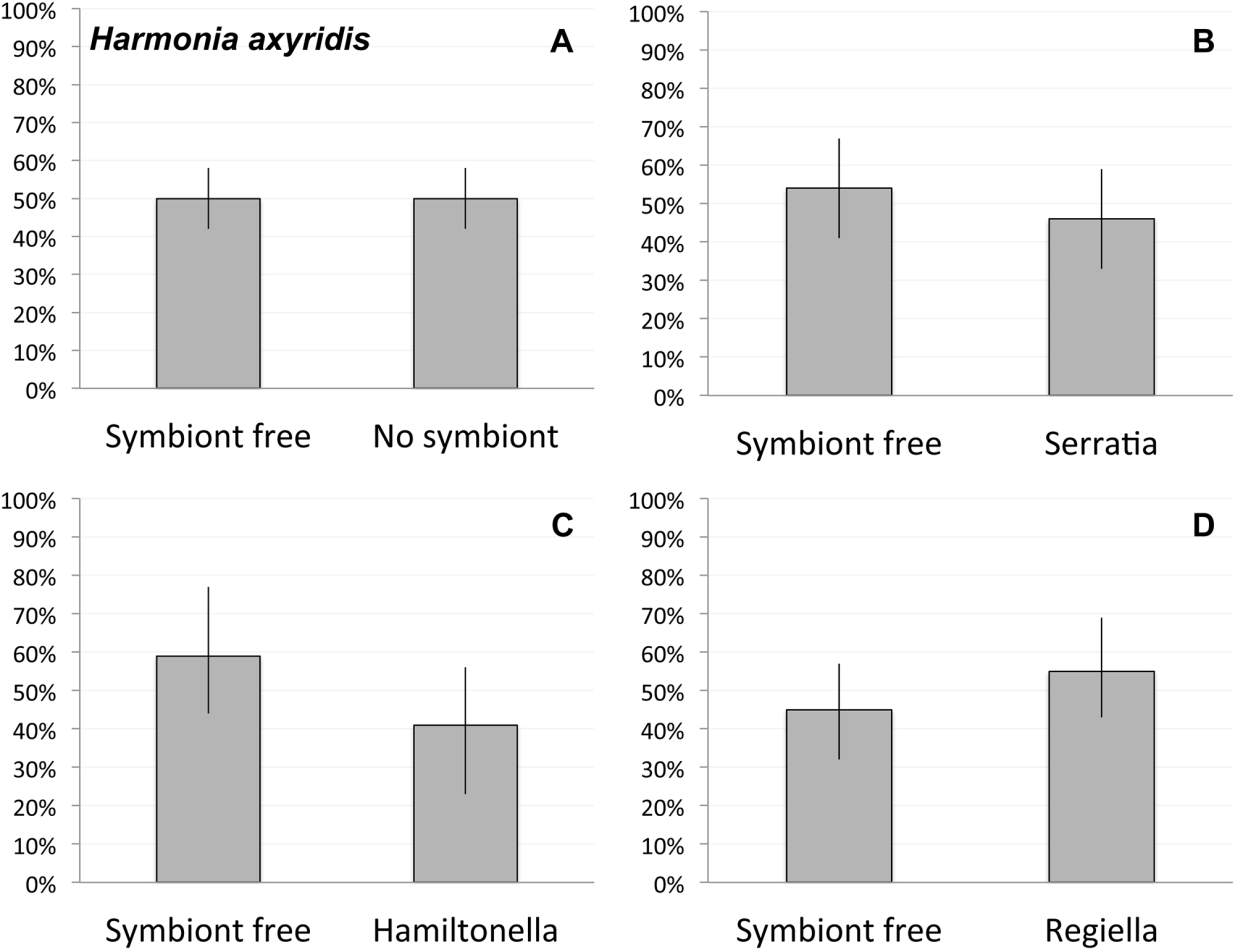
Results of aphid symbiont choice experiments in *Ha*. *axyridis*. **A)** Observed percentages of aphids chosen for all 134 *Ha*. *axyridis* trials with symbiont types pooled. Observed percentages of aphids chosen in the **B)** 52 trails with the *Serratia* symbiont, **C)** the 53 trials with the *Regiella* symbiont, and **D)** 29 trials *Hamiltonella* symbiont. The bars represent the 95% confidence interval for the observed percentages.

In both species, we found no preference for symbiont free aphids in any of the trials. Adult *Ha*. *axyridis* were just as likely to approach aphids with symbionts as those without (Fig 1A, *χ*^2^ = 0.0, *p* = 1.0). In the 134 total trials performed with *Ha*. *axyridis* and either single aphids or five aphids in the choice chambers, we found that the lady beetles approached the cage with the symbiont free aphid in about half of the trials and the aphid with one of the three secondary symbionts in half of the trials (Fig 1B–1D, *Serratia/* symbiont free: *χ*^2^ = 0.31, *p* = 0.58; *Regiella/* symbiont free: *χ*^2^ = 0.47, *p* = 0.49; *Hamiltonella/* symbiont free: *χ*^2^ = 0.86, *p* = 0.35). In the 70 single aphid trials, we found no difference from the expected 50% choice for *Serratia* (*Serratia/* symbiont free: *χ*^2^ = 0.03, *p* = 0.85), *Regiella* (*Regiella/* symbiont free: *χ*^2^ = 1.40, *p* = 0.24), or *Hamiltonella* (*Hamiltonella/* symbiont free: *χ*^2^ = 0.60, *p* = 0.44) versus aphids uninfected by secondary symbionts. For the subset 64 *Ha*. *axyridis* trials that used five aphids per mesh cage, we again found no difference from the expected 50% choice for *Serratia* (*Serratia/* symbiont free: *χ*^2^ = 1.10, *p* = 0.30), *Regiella* (*Regiella/* symbiont free: *χ*^2^ = 0.04, *p* = 0.85), or *Hamiltonella* (*Hamiltonella/* symbiont free: *χ*^2^ = 0.29, *p* = 0.59) versus aphids uninfected by secondary symbionts. The results from the one aphid and five aphid trials were not significantly different from each other for any symbiont group (*Serratia*: *χ*^2^ = 0.82, *p* = 0.36; *Regiella*: *χ*^2^ = 0.96, *p* = 0.33; *Hamiltonella*: *χ*^2^ = 0.02, *p* = 0.87). Overall these results suggest that *Ha*. *axyridis* lady beetles are not avoiding aphids with symbionts.

**Fig 2:**
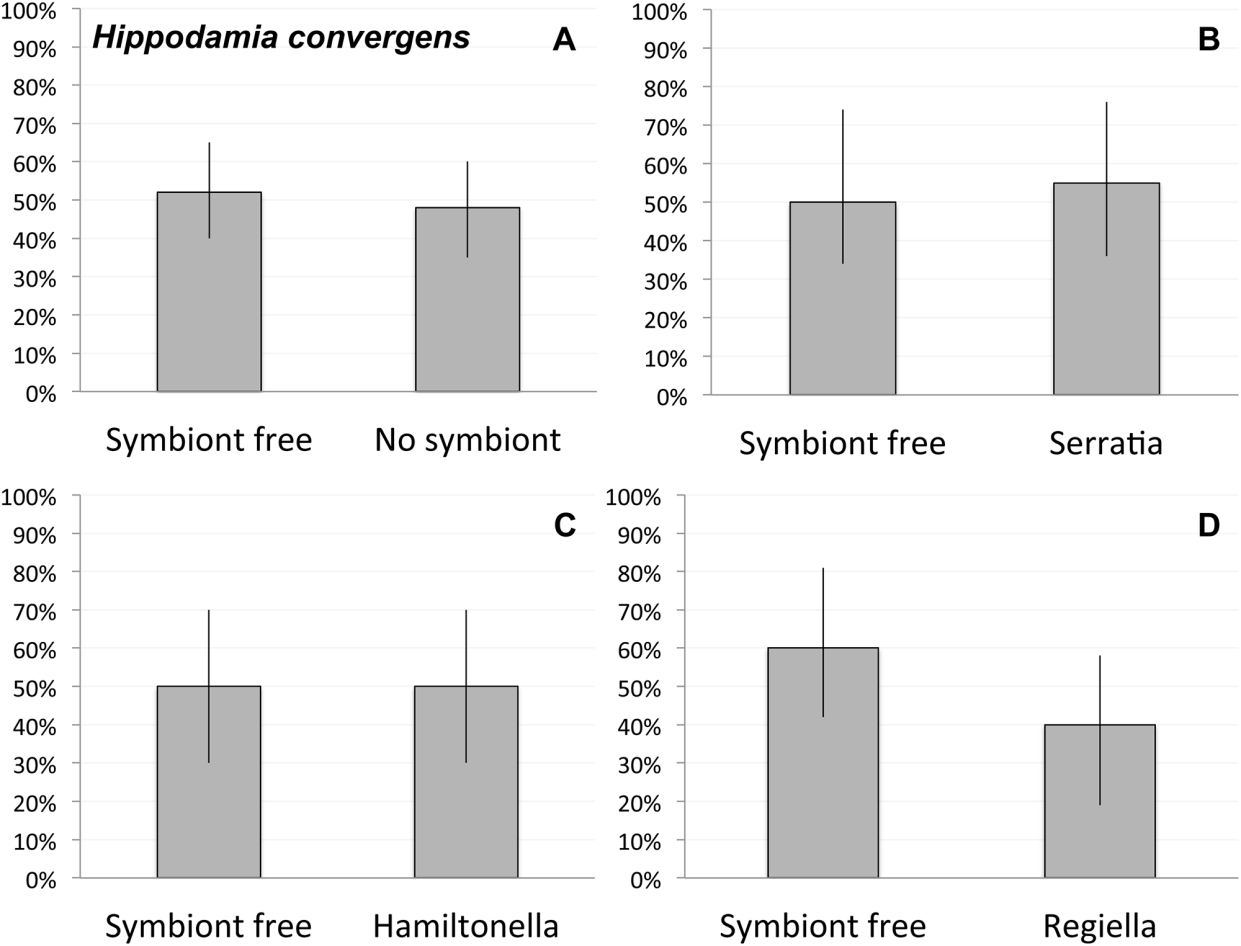
Results of aphid symbiont choice experiments in *Hi*. *convergens*. **A)** Observed percentages of aphids chosen for all 60 *Hi*. *convergens* trials with symbiont types pooled. Observed percentages of aphids chosen in the 20 trails with the **B)** *Serratia* symbiont, **C)** *Regiella* symbiont, and **D)** *Hamiltonella* symbiont. The bars represent the 95% confidence interval for the observed percentages.

In the 60 total *Hi*. *convergens* trials run, we found no evidence for any avoidance or choice based on aphid symbiont status. Adult *Hi*. *convergens* were just as likely to approach aphids with symbionts as those without (Fig 2A, *χ*^2^ = 0.07, *p* = 0.80). For each of the aphid symbiont types, we would expect the lady beetles to approach the symbiont free aphids in 50% of the trials and the aphids with one of the three symbiont types in 50% of the trials by chance alone. Our results were not significantly different from these expected results (Fig 2B–2D, *Serratia/* symbiont free: *χ*^2^ = 0.20, *p* = 0.65; *Regiella/* symbiont free: *χ*^2^ = 0.81, *p* = 0.37; *Hamiltonella/* symbiont free: *χ*^2^ = 0.0, *p* = 1.0) suggesting that as in *Ha*. *axyridis*, adult lady beetles do not discriminate between aphids with or without symbionts.

There were no differences between males in females in their behavior. In both species, both males and females were equally likely to choose symbiont harboring aphids as symbiont free aphids (*Ha*. *axyridis*: *Serratia*: *χ*^2^ = 1.35, *p* = 0.25; *Regiella*: *χ*^2^ = 0.46, *p* = 0.50; *Hamiltonella*: *χ*^2^ = 0.83, *p* = 0.36; *Hi*. *convergens*: *Serratia*: *χ*^2^ = 0.31, *p* = 0.58; *Regiella*: *χ*^2^ = 0.35, *p* = 0.55; *Hamiltonella*: *χ*^2^ = 0.97, *p* = 0.33). Additionally, there was no bias towards the left or right mesh chambers in any of the trials in either species (*Ha*. *axyridis*: *χ*^2^ = 1.47, *p* = 0.23, *Hi*. *convergens*: *χ*^2^ = 3.01, *p* = 0.39). Finally, because we used the same ten individuals in all the *Ha*, *axyridis* trials, we were also able to see whether individuals showed any preferences for specific aphid types or directions. We found no evidence for individual preference for either aphid symbiont type ($\chi_{27}^{2}$ = 25.70, *p* = 0.54) or for a particular direction (left or right; $\chi_{9}^{2}$ = 5.53, *p* = 0.79). Additionally, for *Ha*. *axyridis* in the first four trials for all individuals beetles were presented with aphids without symbionts and those harboring the *Regiella* symbiont. We saw no evidence of trial number on aphid choice for the first four trials ($\chi_{3}^{2}$ = 1.75, *p* = 0.63) All relevant data is available in the supporting information files (S1 File).

## Discussion

Overall, we found no evidence to support our prediction that the adults of two species of lady beetle choose aphids without secondary symbionts over those with *Serratia*, *Regiella*, or *Hamiltonella* symbionts using our experimental set-up. In all of the trials conducted, focal lady beetles were just as likely to move towards and remain near cages containing aphids with symbionts as those containing aphids without symbionts, and our results were no different than if direction were determined by chance alone. We do note that for choice experiments with *Hi*. *convergens*, based on our sample size, we only have sufficient power to detect significance if there were large effects of symbiont on choice (Cohen 1977). Though, we did have enough power in our experiments with *Ha*. *axyridis* to detect medium size effects as well, and still did not see a significant difference from 50/50 for those choice experiments (Cohen 1977). Additionally, previous work done in *Hi*. *convergens* adult male and female lady beetles found no significant differences in feeding rates between pairs fed aphids without symbionts and those fed aphids with either the *Hamiltonella* or *Serratia* facultative symbiont. Adult male and female *Hi*. *convergens* ate just as many symbiont harboring aphids as symbiont free aphids over a seven day period [13]. Our *Ha*. *axyridis* were wild caught, so we do not know what their previous experience with aphid symbionts was prior to our choice experiments. Our *Hi*. *convergens* adults were provided by a biological supply company, and again we do not know if there adults were naïve or had previous experience with aphid harboring symbionts. Finally, we cannot rule out that lady beetles may also decide to avoid eating aphid prey with symbionts through mechanisms such as biting or partially eating an aphid, an act which was not possible in our experimental set-up.

Interestingly, while our results do not support the prediction that lady beetles avoid or prefer aphids with or without symbionts, there may be some symbiont-related aphid behavioral differences that could result in differential lady beetle predation rates. Polin et al. (2014) reported that while the lady beetle *Adalia bipunctata* was just as likely to attack aphids with or without symbionts, aphid symbiont infection status affected predator success rates. Behavioral observations revealed that the difference in predation rate was due to higher levels of defensive evasion in aphids without facultative symbionts than those with multiple symbionts (*Rickettsiella* and *Hamiltonella*), suggesting that symbiont status may alter aphid predator avoidance behavior and therefore affect host fitness in negative way [22]. This suggests that while symbionts may directly protect aphids from parasitism and heat shock [3], they may also alter aphid behavior in ways that may have negative fitness effects for their hosts.

Symbionts may also alter the physical traits of their aphid hosts, and those changes could affect their susceptibility to predation. For example, red pea aphids harboring the *Rickettsiella viridis* symbiont turn green as they age. *Rickettsiella* harboring green aphids were less likely to be predated upon by the lady beetle *Coccinella septempunctata* than red aphids without symbionts, perhaps due to these symbiont induced differences in host coloration [23]. However, symbiont-induced differences in the chemical composition of the aphid alarm pheromone E-β-farnesene (EBF) could also affect the foraging behavior and foraging efficiency of aphid predators [6], thereby providing another way in which symbionts can affect the interactions between hosts and their natural enemies.

Our previous work found that in *Hi*. *convergens* larvae fed aphids with either the *Hamiltonella* or *Serratia* symbionts were two times more likely to die before reaching adult emergence [13]. In *Ha*. *axyridis* larvae that were fed aphids with the *Serratia* or *Regiella* symbionts were nine and seven times more likely to die prior to reaching pupation, respectively, than those fed aphids without secondary symbionts [14]. Despite these potential negative fitness consequences in the larvae, we found no evidence of avoidance in adult lady beetles in either species. This could perhaps be due to the absence of fitness effects in the adults. If only larvae experience negative fitness effects, than we would not expect adult lady beetles to avoid aphids with symbionts. Additionally, this may be due to the constraints of our choice chambers which only allowed for limited contact between the aphids and lady beetles and may not have allowed the lady beetles to properly assess the aphid. The aphid parasitoid wasp *Aphidius ervi* does appear to be able to discriminate between aphids with the *Hamiltonella* symbiont and those without it [6]. Wasp eggs laid in aphids harboring *Hamiltonella* and the *Hamiltonella* associated APSE phage fail to develop [15]. Superparasitism, the laying of more than one egg at a time, can result in higher rates of successful parasitism for wasps that lay eggs in aphids with *Hamiltonella*. *Aphidius ervi* wasps are significantly more likely to superparasitize aphids with *Hamiltonella* than those without the symbiont [6]. Again, there are also differences in the composition of the aphid alarm pheromone EBF which could provide a basis for wasps to discriminate between aphid types [6]. These volatile chemical signals could also potentially be used by other aphid predators.

All of the current work done to understand the effects of aphid symbionts on lady beetle survival and behavior have been done in the lab. Within natural aphid populations, we know that there is variation in symbiont infection status and that the percentage of aphids harboring symbionts can change significantly and frequently within a single summer [2,10,24,25]. This suggests that within natural populations there are changes in the costs and benefits of harboring secondary symbionts over time [26] and that a variety of factors, including multiple natural enemies, temperature, and host plant availability can affect aphid-symbiont dynamics. Ultimately, this variation may minimize the fitness effects of aphid symbionts on lady beetles and therefore reduce selection for avoidance behavior in these species. Therefore it is crucial that we consider the potential impact of symbionts across food webs in ecologically realistic field studies. Future work should extend beyond the lab to better understand these potentially complex interactions in natural field populations [26,27].

## Supporting information

S1 File Raw data. All raw data used in the analyses described in this manuscript.
(XLSX)
